# Changes in salivary biomarkers associated with periodontitis and diabetic neuropathy in individuals with type 1 diabetes

**DOI:** 10.1038/s41598-022-15430-0

**Published:** 2022-07-04

**Authors:** Larissa Steigmann, Shogo Maekawa, Frederic Kauffmann, Jacob Reiss, Ashley Cornett, James Sugai, Julian Venegas, Xudong Fan, Yuying Xie, William V. Giannobile, Rodica Pop-Busui, Isabelle M. A. Lombaert

**Affiliations:** 1 Department of Periodontics and Oral Medicine, School of Dentistry, University of Michigan, Ann Arbor, MI 48106 USA; 2 Department of Periodontology, Tokyo Medical and Dental University, Tokyo, 113-8510 Japan; 3 Department of Oral and Craniomaxillofacial Surgery, Center for Dental Medicine, University Medical Center Freiburg, 79106 Freiburg Im Breisgau, Germany; 4 Division of Metabolism, Metabolism Endocrinology and Diabetes, Department of Internal Medicine, School of Medicine, University of Michigan, Ann Arbor, MI 48105 USA; 5 Department of Biologic and Materials Sciences, School of Dentistry, University of Michigan, Ann Arbor, MI 48109 USA; 6 Biointerfaces Institute, University of Michigan, Ann Arbor, MI 48109 USA; 7 Department of Computational Mathematics, Science and Engineering, Michigan State University, East Lansing, MI 48824 USA; 8 Department of Biomedical Engineering, College of Engineering, University of Michigan, Ann Arbor, MI 48109 USA; 9 Present Address: Department of Oral Medicine, Infection, and Immunity, Harvard School of Dental Medicine, Boston, MA 02115 USA

**Keywords:** Biomarkers, Predictive markers, Diseases of the nervous system, Neurological disorders, Diabetes, Diabetes complications, Type 1 diabetes

## Abstract

The objective of this pilot clinical study was to identify salivary biomarkers that are associated with periodontal disease and measures of diabetic autonomic dysfunction. Saliva samples from 32 participants were obtained from 3 groups: healthy (H), type 1 diabetes mellitus (DM), and type 1 diabetes mellitus with neuropathy (DMN). Based on the periodontal examination, individuals’ mean Periodontal Screening and Recording scores were categorized into two groups (periodontally healthy and gingivitis), and correlated to specific salivary inflammatory biomarkers assessed by a customized protein array and enzyme assay. The mean salivary IgA level in DM was 9211.5 ± 4776.4 pg/ml, which was significantly lower than H (17,182.2 ± 8899.3 pg/ml). IgA in DMN with healthy periodontium was significantly lower (5905.5 ± 3124.8 pg/ml) compared to H, although IgA levels in DMN patients with gingivitis (16,894. 6 ± 7084.3) were not. According to the result of a logistic regression model, IgA and periodontal condition were the indicators of the binary response given by H versus DM, and H versus DMN, respectively. These data suggest that selected salivary biomarkers, such as IgA, combined with a periodontal examination prior to obtaining salivary samples can offer a non-invasive method to assess risk for developing diabetic neuropathy.

## Introduction

Diabetic neuropathies are the most prevalent chronic complications of diabetes^1^. Up to 20% of individuals with newly diagnosed diabetes, particularly type 2 diabetes, may present with neuropathy, and its prevalence in long-standing individuals with diabetes is above 50%^1–4^. Neuropathies affect sensory, motor and autonomic nerve fibers in any part of the body. The disease is characterized by progressive damage and loss in all populations of peripheral nerve fibers and neurons, demyelination, impaired nerve regeneration, and ultimately nerve fiber dysfunction^1,5^. The clinical spectrum of diabetic neuropathies is broad, although by far distal symmetrical polyneuropathy and autonomic neuropathy are most prevalent^1^. No disease-modifying treatments are currently available that target damage to autonomic nerve fibers, thus, a timely diagnosis of the earliest stages of diabetic autonomic neuropathy is warranted to initiate prevention and interventions^1^. Unfortunately, given the anatomical distribution of the autonomic nerves, a sensitive and non-invasive diagnosis tool to detect early nerve fiber damage in individuals with diabetic autonomic neuropathy is still unavailable^6^, highlighting the urgent need for early diagnosis tools through correlative biomarkers.

Diabetic autonomic neuropathy affects both the autonomic parasympathetic and sympathetic neurons. The autonomic nervous system innervates multiple organs, and one example is the salivary glands where it regulates salivary flow and various secreted enzymes, proteins, and immunoglobulins^7,8^. Hence, alterations in the ratio of parasympathetic and sympathetic innervation, such as post-injury, can lead to a dysregulation in organ function and saliva secretion^9–13^. Salivary biomarkers have been used in diagnostic tools for various diseases, including viral infections, malignancies, and diabetes^14^. Specifically, Immunoglobin A (IgA) and alpha-Amylase (α-Amylase) have been examined to identify diabetic conditions without invasive examinations. Thus, salivary samples may serve as potential detection tools for identifying changes in the sympathetic/parasympathetic balance during early stages of diabetic autonomic neuropathy and its progression.

Periodontal disease is emerging as a risk factor for the development of chronic complications in patients with diabetes^15,16^, and its role in the pathogenesis of diabetic neuropathy has been suggested in recent reports^17,18^. Periodontal disease is mainly attributed to a bacterial challenge that leads to inflammation with further breakdown of the soft and hard tissues surrounding the teeth. This inflammatory response will not only occur locally in the oral cavity, but circulating inflammatory mediators will also lead to a systemic response^19^. The latter will worsen the already occurring microvessel damage due to chronic hyperglycemia, and thus, periodontal disease should be co-evaluated when analyzing oral-based biomarkers.

In this study, we hypothesized that an approach combining measures of periodontal disease and salivary analytes can be used as early biomarkers to detect earlier imbalances in the autonomic nervous system heralding diabetic neuropathy. Thus, the primary goal is to identify oral-based biomarkers that uniquely correlate with diabetic autonomic neuropathy that serve as predictors for development of neuropathy.

## Results

### Characteristics of the subjects

Ten H, ten DM, and twelve DMN participants were enrolled and completed all evaluations described for data analysis. One female participant in the DM group was excluded from further analysis given that her age fell outside the age match. The characteristics of the participants with valid data are shown in Table 1.

**Table 1:** Demographic and periodontal parameters of the three experimental groups.

Demographist and clinical characteristics	H	DM	DMN
N	10	9	12
Age (yrs)	40 ± 12	44 ± 16	56 ± 11*
**Sex**			
Male	4	4	10
Female	6	5	2
**Smoking**			
Current smoker	0	0	0
Past smoker	1	4	2
Never smoker	8	5	7
Missing	1	0	3
Weight (kg)	71.6 ± 14.8	84.5 ± 22.5	91.6 ± 12.5*
Height (m)	1.72 ± 0.13	1.72 ± 0.10	1.75 ± 0.06
BMI	24.0 ± 2.8	28.0 ± 5.0	29.9 ± 4.6*
HbA1c (%)	5.4 ± 0.1	8.3 ± 2.3	8.4 ± 1.6*
Fasting Blood Glucose level (mg/dL)	85.0 ± 10.1	173.6 ± 92.2	153.6 ± 72.1
Duration of Disease (years)	0	16 ± 7**	34 ± 15**,^††^
Amounts of Smoking (pack/year)	0.11 ± 0.33	0.43 ± 1.1	3.13 ± 8.84
Mean PSR Value	0.30 ± 0.42	0.85 ± 0.88	1.00 ± 0.99
**Salivary biomarkers**			
α-Amylase level (U/mL)	104.0 ± 91.8	207.5 ± 174.0	130.9 ± 112.4
CRP (pg/mL)	763.1 ± 799.2	911.0 ± 970.2	770.7 ± 358.4
IgA (pg/mL)	17,182.2 ± 8899.3	9211.5 ± 4776.5*	11,389.9 ± 7360.3
IL-10 (pg/mL)	6.5 ± 7.7	10.2 ± 24.2	8.5 ± 13.5
IL-6 (pg/mL)	409.6 ± 493.5	943.2 ± 1357.1	894.3 ± 905.1
NFkB (pg/mL)	2423.4 ± 1602.5	2846.7 ± 2800.4	6155.7 ± 6755.8
TNF-α (pg/mL)	1893.4 ± 3027.6	4271.5 ± 9982.6	3357.1 ± 5099.7

Sociodemographic parameters and saliva biomarker levels are shown. Data are presented as mean ± standard deviation. Tukey test (for continuous data) and/or Chi-square test (for categorical data) were used to assess differences between groups. a) Significantly different from H, *p < 0.05, **p < 0.01. b) Significantly different from DM, ^††^p < 0.01. H: Healthy subjects, DM: individuals with diabetes without neuropathy, DMN: individuals with cardiovascular autonomic diabetic neuropathy, N: sumple numbers, BMI: Body Mass Index, PSR: Periodontal Screening Record, CRP: C-reactive protein, IgA: Immunoglobulin A, IL: Interleukin, NFκB: nuclear factor kappa-light-chain-enhancer of activated B cells, TNF: Tumor Necrosis Factor.

Briefly, the H and DM individuals were age-and sex-matched. Individuals with DMN were older (40 ± 12, 44 ± 16, and 56 ± 11 years old in H, DM, and DMN, respectively), more likely to be women, and having a longer diabetes duration compared to DM (34 ± 15 years vs 16 ± 7 years p <  < 0.001). Hemoglobulin A1c (HbA1c), body mass index (BMI) and body weight were higher in the DMN group, and there was a nonsignificant trend for more smokers in DMN. There were no other significant differences in participant’s characteristic (Table 1).

Next, a non-metric multi-dimensional scaling was used to make a prediction analysis of patient characteristics between groups. No variances were observed between the group cohorts (Supplementary Fig. 1A and B). The logistic regression model identified age, BMI and duration as significant risk factors for DMN (Table 2).

**Table 2:** Predictors analysis with the Bayes Logistic Regression.

	H vs DM		H vs DMN		DM vs DMN	
	Coefficient estimate ± SEM	p-value	Coefficient estimate ± SEM	p-value	Coefficient estimate ± SEM	p-value
**Sociodemographic**						
Age	0.022 ± 0.031	0.476	0.092 ± 0.039	0.016*	0.052 ± 0.032	0.104
Sex	−0.146 ± 0.833	0.861	−1.683 ± 0.888	0.058	−1.506 ± 0.900	0.094
BMI	0.210 ± 0.118	0.191	0.321 ± 0.127	0.012*	0.071 ± 0.088	0.191
Duration of Disease					0.114 ± 0.050	0.022*
**Biomarkers in saliva**						
α-Amylase	0.005 ± 0.004	0.256	0.002 ± 0.004	0.57	−0.002 ± 0.004	0.526
CRP	0.000 ± 0.001	0.653	0.000 ± 0.001	0.979	0.001 ± 0.001	0.399
IgA	0.000 ± 0.000	0.048*	0.000 ± 0.000	0.151	0.000 ± 0.000	0.393
IL-10	−0.135 ± 0.108	0.212	0.015 ± 0.038	0.701	0.145 ± 0.126	0.25
IL-6	0.000 ± 0.001	0.596	0.001 ± 0.001	0.199	0.001 ± 0.001	0.369
NF-κB	0.000 ± 0.000	0.604	0.000 ± 0.000	0.192	0.000 ± 0.000	0.136
TNF-α	0.000 ± 0.000	0.508	0.000 ± 0.000	0.474	0.000 ± 0.000	0.279
**Periodontal condition**						
PSR category	2.498 ± 1.562	0.11	3.423 ± 1.605	0.033*	0.711 ± 0.832	0.392

Each row indicates the predictor of the binary response, given by each column (e.g. healthy vs diabetes), using a logistic regression model. *p < 0.05.

### Periodontal condition

**Figure 1:**
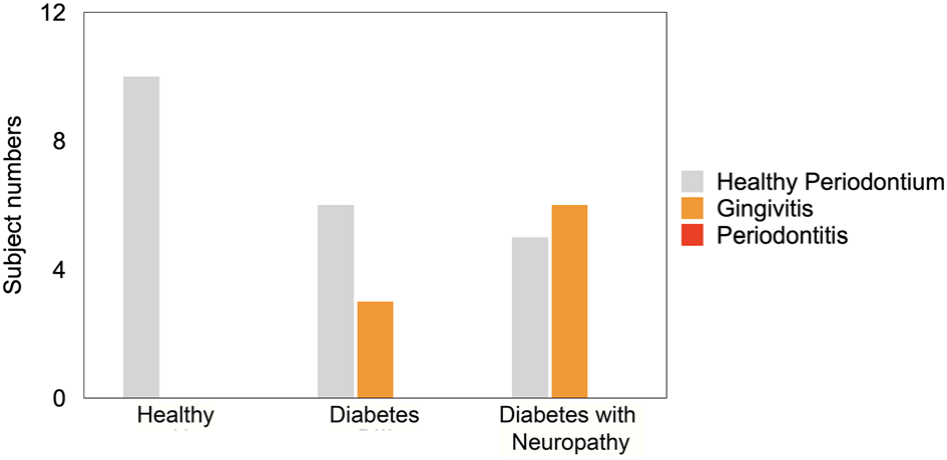
Periodontal profiles among the groups. PSR category-0: Healthy Periodontium, PSR-1: Gingivitis, PSR-2: Periodontitis. Chi-square test, p < 0.05.

Although no participants presented with a definite diagnosis of periodontitis defined as PSR > 1 (Fig. 1), 100% of the H participants presented with good oral health (healthy periodontium, PSR of 0) compared to 66% of DM and only 42% of DMN patients. Gingivitis (PSR of 1) presented in 33% and 58% of the DM and DMN group, respectively (p < 0.05). When analyzing the PSR as a continuous variable, we found a trend for higher values in DM and DMN groups compared to H (Table 1). Gingivitis (PSR 1) was also observed to be more likely associated with DMN compared to H (Table 2). In addition, HbA1c was significantly higher in DM with gingivitis, compared to DM with healthy periodontium (Supplementary Table 2).

### Salivary biomarkers

**Figure 2:**
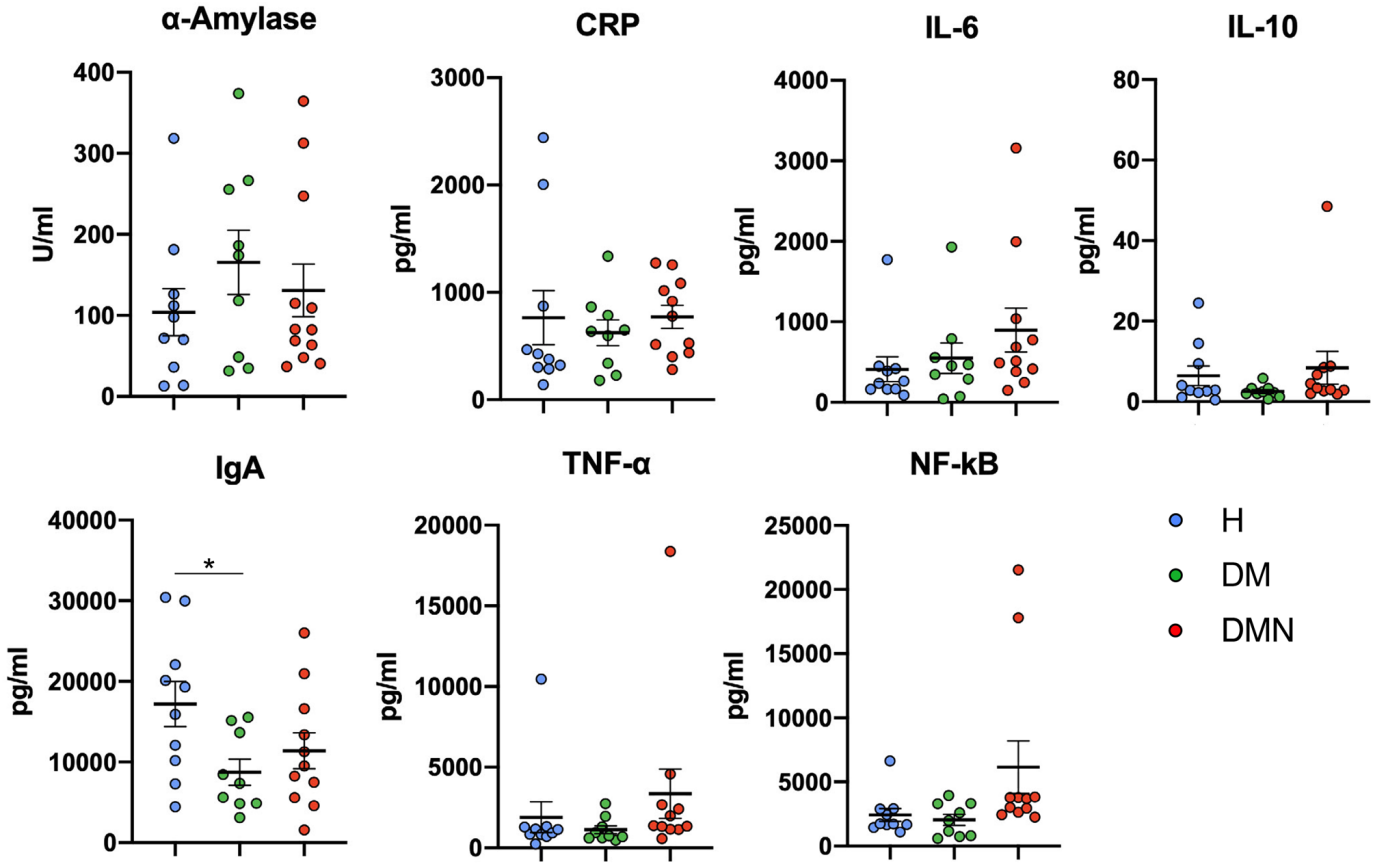
Biomarker levels in saliva among the groups. Mean ± SEM. One-way ANOVA and Tukey were performed. *p < 0.05.

We next compared the salivary biomarkers analyzed among groups. Global comparison of mean values showed significantly lower IgA levels in saliva in the DM group compared to H (p < 0.05) (Table 1, Fig. 2). In addition, trends of higher levels in α-amylase level and IL-6 were observed in the DM versus H group. Although no significant differences were noted between DMN and DM, there was a trend for decreased levels of saliva α-amylase in the DMN group. In contrast, IL-6, TNF-α and NF-κB showed a trend for increasing levels. Once a Bayes general linear model was performed to distinguish indicators for disease, lower salivary IgA appeared as a significant indicator for DM versus H (p < 0.05) (Table 2).

**Figure 3:**
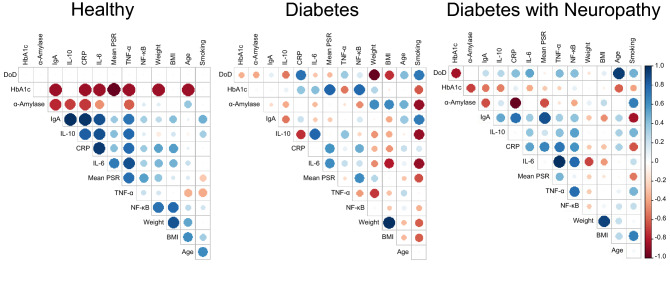
The heatmap patterns with spearman correlation coefficient. Each row and column show biomarkers and clinical parameters. The colors represent coefficient values (blue means positive correlation and red means negative), and the circle sizes are scaled by the correlation magnitudes.

To outline correlations between the various biomarkers and participants’ characteristics, we created a correlative heatmap using a Spearman correlation coefficient (Fig. 3). Herein, various trends in these biomarkers were observed from H towards the DM and DMN stage. In Fig. 3, the negative correlations between HbA1c and biomarkers observed in H tended to be neutral or reversed in the DM and DMN groups. Moreover, there was an increasing correlation between IL-10/α-amylase from H to DM and DMN, a decreasing α-amylase/CRP correlation between DM and DMN, and many other increasing correlations between DM and DMN (e.g., IL-10/IgA) (Supplementary Fig. 2). In addition, IgA showed a significant negative correlation with α-amylase in H participants, which was attenuated in the DMN group, but neutral in DM. (Supplementary Fig. 3).

In the next step, we analyzed the salivary biomarkers according to participants’ group and healthy periodontium or gingivitis status (Supplementary Table 2). As expected, the mean PSR was significantly higher in the DM/DMN group compared to H. From all saliva biomarkers, IgA was significantly lower in DMN with healthy periodontium compared to H (p < 0.05). Although it was not significant, the IgA level in DM with healthy periodontium was also lower than H, but higher than DMN with healthy periodontium. As such, IgA could serve as a potential biomarker in patients with healthy periodontium wherein declining IgA levels in saliva can predict a progression from the H towards DMN stage.

While other analytes were not significant amongst the sub-groups, there was a trend of lower α-amylase, higher IL-10, IgA, and TNF-α in the DMN with gingivitis group as compared to DMN with healthy periodontium (Supplementary Table 2). When comparing DMN with healthy periodontium to DM with healthy periodontium, there was a trend in higher IL-6 and NF-κB levels. In contrast, DMN patients with gingivitis showed a trend in higher levels of IL-10, TNF-α, IgA and NF-κB while α-amylase was lower. Overall, these data suggest that PSR status plays an essential role when comparing salivary biomarkers between H, DM and DMN patients.

## Discussion

In this pilot study we evaluated salivary biomarkers and periodontal disease in type 1 diabetes/neuropathy patients, and found a significantly worse gingival health in participants with type 1 diabetes/neuropathy compared with age-matched healthy participants. More importantly, a significant gradual decrease of salivary IgA was observed from healthy individuals towards patients with type 1 diabetes and type 1 diabetes with neuropathy. Hence, this was only present in neuropathy patients with healthy periodontium as IgA was significantly higher in participants presenting both gingivitis and neuropathy. To the best of our knowledge, this is a previously un-described link in type 1 diabetes patients progressing with neuropathy.

To date, evidence regarding salivary IgA in diabetes patients is limited and inconclusive. Some studies report significantly higher levels of salivary IgA in diabetes patients compared to those without^20^, while others demonstrated lower salivary IgA secretion rates^21^ and concentrations in patients with diabetes^22^. The latter observation is in line with our finding that specifically focuses on type 1 diabetes patients progressing towards neuropathy and presenting a healthy periodontium. As secretory IgA is the predominant immunoglobulin in secretions of the mucosal immune system, less IgA production could cause a problem for those patients as subjects with selective IgA deficiencies have higher prevalence of infectious disease and/or precipitate to autoimmune disease^23^. On the other hand, the severity of gingivitis is positively correlated with IgA levels^24^, thus it is plausible that higher levels of IgA in participants with diabetic neuropathy and gingivitis are the result of gingival inflammation itself, which becomes more severe with advanced diabetes^25,26^.

Existing data on other salivary analytes in individuals with diabetes, particularly with neuropathy, is also limited. Given the positive correlation between blood glucose and serum HbA1c with glucose levels found in saliva of diabetes patients^27^, several efforts have been directed to use saliva as a biomarker analysis tool. Despite saliva collection is less invasive than blood, the levels of glucose are lower, and thus, measurements are associated with higher costs^28,29^. Besides glucose, other saliva analytes (e.g. leptin, insulin, nerve growth factor, IL’s, TNF-α, hepatocyte growth factor, common salivary protein 1, cortisol, lactic acid) have been explored in relationship with diabetes^27,30–32^, but often provided somewhat conflicting findings due to their various study designs and inclusion of both type 1 and 2 adult diabetic patients. For example, α-amylase enzyme levels were described as either higher^22,33^, similar^34^, or decreased^35^ in diabetic patients in comparison to healthy individuals. Our adult type 1 diabetic study cohort demonstrated an increased trend in α-amylase, which extends results found in type 1 diabetic children^36^. We also found an increased trend of CRP and HbA1c in type 1 adults, which reflects data from individuals with type 2 diabetes^37^ and type 1 pediatric patients (HbA1c data only)^38^. It should be noted that salivary flow rates are often found as lower in individuals with type 1 diabetes compared to healthy, and thus, this can influence the overall concentration of biomarkers in saliva^39,40^.

We also assessed the relationship of salivary enzyme α-amylase, IgA, as well as other biomarkers and patient characteristics. We found a significant negative correlation between IgA and α-amylase in healthy patients that neutralized in diabetic patients, but regressed back in diabetic patients with neuropathy. Both salivary IgA and α-amylase are largely under autonomic nerve control. Salivary IgA can be stimulated by both the parasympathetic and sympathetic nerve system^11,12^ while α-amylase is majorly under sympathetic control^13^, suggesting changes in the IgA/α-amylase ratio could reflect alterations in the autonomic nervous system when progressing towards type 1 diabetes and/or neuropathy. Thus, alterations in the salivary enzymatic compounds, and correlations thereof, could serve as diagnostics for monitoring early stage progression of diabetes-induced neuronal damage.

There is abundant evidence demonstrating that certain inflammatory biomarkers are elevated for years prior to resulting into clinically significant consequences^41^. Previous investigators have analyzed salivary biomarkers’ status to correlate to the patients’ medical status^42^. However, there have been no studies yet that linked inflammatory biomarkers CRP, IL-6, IL-10, TNF-α, and NF-κB in saliva from adult individuals with type 1 diabetes with and without microvascular complications. The pro-inflammatory cytokines TNF-α^25,43^, IL-6 ^44,45^, and NF-κB^46^ are frontrunner biomarkers for worsening periodontal conditions, and are also potent proinflammatory cytokines involved in the pathogenesis of diabetic neuropathy. In congruence with separately reported pathological mechanistic roles, we observed increased trends of inflammatory cytokine levels in the neuropathy with gingivitis group compared to neuropathy patients with healthy periodontium. Caseiro et al. identified content of some specific protein fragments, known to be related with bacterial attachment, that eventually leads to accumulation of periodontal pathogens and gingival inflammation^47^. Indeed, the severity of inflammatory periodontal disease increases with the presence of diabetic complications^48^. Thus, our data confirms the broader relationship between type 1 diabetes mellitus/diabetic induced neuropathy and PSR by demonstrating a worsening of the periodontal status from healthy over diabetic conditions to diabetes induced neuropathy. These findings should be interpreted in the framework of the bidirectional relationship between periodontal disease and diabetes mellitus, and periodontal disease being common a complication of diabetes mellitus^49^.

While our study is one of a kind to correlate saliva biomarkers in a type 1 diabetic cohort with local inflammation in periodontal tissue, the sample number in the study is small and larger studies are needed to confirm our results. We recognize that the current outcomes of our study are of descriptive nature and present lack of power due to limited samples per study group. Furthermore, the chosen analytes were determined by the study group to test our hypothesis, and hence are a source of bias. A global saliva proteomic analysis^38^ could reduce this bias, and a more in depth periodontal examination could also be executed in the future.

Overall, current literature presents several controversies on saliva biomarkers, as some studies only included diabetic patients with significantly worse oral conditions^20^, lacked descriptions about the type of diabetes^21^, or only compared type 2 DM with healthy subjects^22^. Our study uniquely reports a set of data solely from a type 1 diabetic cohort with and without neuropathy, providing an opportunity to potentially distinguish masked biomarker observations found in type 1 and 2 mixed cohorts. As a result, this proof-of-concept study contributes to the emerging literature to enhance diagnostics of salivary biomarkers, in combination with the patient’s periodontal condition, as preventative tools in the field of diabetes and neuropathy.

## Conclusion

The early recognition and appropriate management of diabetic neuropathy is crucial, as early detection of neuropathy may improve symptoms, reduce sequelae, and improve quality of life. Changes in the salivary biomarkers, such as IgA, could serve as diagnostic and monitoring tools to identify diabetes-induced tissue damage. IgA could be affected by oral inflammatory conditions, thus the significance of a combined periodontal tissue examination prior to obtaining salivary samples is crucial to the predictive evaluation and even more so, larger studies have to be conducted to confirm its role. Our findings contribute to early disease intervention strategies and highlight the potential use of saliva for the monitoring diabetes-related neuropathy with concomitant periodontal inflammation among individuals with type 1 diabetes.

## Methods

### Ethics Statement

This study was approved by the Institutional Review Board (IRB) at the University of Michigan (IRB#HUM00143679, Lombaert). All participants provided written informed consent prior to participation in the study, and all methods were performed in accordance with the relevant guidelines and regulations.

### Study design and participants

This was a cross-sectional pilot study in men and women with diabetes and age-matched healthy controls. Thirty-two human volunteers participated in this study. Inclusion and exclusion criteria are presented in Supplementary Table 1. According to these criteria, subjects were classified as (1) healthy (H), (2) individuals with diabetes without neuropathy (DM), and (3) individuals with cardiovascular autonomic diabetic neuropathy or mild-to-moderate peripheral neuropathy (DMN). Cardiovascular autonomic diabetic neuropathy (CAN) was documented with the gold standard cardiovascular autonomic reflex tests (CARTs) and heart rate variability studies, as previously described^50^. All individuals received a complete periodontal examination, and saliva was collected for experimental purposes.

### Periodontal examination

Clinical periodontal examination was performed by one calibrated periodontist (FK). The examination consisted of measurements of positive and negative gingival recessions, probing pocket depth from the gingivae, and bleeding on probing at all teeth except for third molars. Clinical parameters were measured at 6 sites per tooth, and included calculus and gingival tissues. Periodontal Screening and Recording (PSR) codes were assigned as follows^51^: code 0 (gingival tissue is healthy with no bleeding on probing), code 1 (bleeding is present on probing), code 2 (supragingival or subgingival calculus and/or defective margins are detected), code 3 (the probing depth is between 3.5 and 5.5 mm), or code 4 (probing depth reaches 5.5 mm or greater). Individual mean PSR score was calculated and subjects were categorized, based on their mean PSR score, as periodontally healthy (PSR 0, mean PSR value was less than 1) or having gingivitis (PSR 1, mean PSR value was between 1 and 2). There were no subjects with a mean PSR value of more than 2, which means that there were no periodontitis patients.

### Saliva collection

Unstimulated whole saliva was collected for each enrolled subject between 9AM and 2PM via the passive drooling technique^52–55^. Individuals were asked to avoid oral hygiene measures, eating, drinking or gum chewing at least 1.5 h prior to saliva collection. All subjects rinsed with tap water (10 mL) for 30 s about 10 min prior to saliva collection and expectorated the water. Next, saliva was collected into sterile tubes for a duration of 15 min or when 2 mL of saliva was collected, whichever came first. Saliva was kept on ice through the entire collection process. Immediately after collection, protease inhibitors (1% Aprotinin, Sigma A6279 and 0.5% Phenyl-methane-sulfonyl fluoride, Sigma P7627) were added. All samples were aliquoted, labeled, and stored at − 80 °C within 30 min of collection.

### Protein microarray and enzyme assay

After thawing, saliva samples were clarified by centrifugation at 12,000 g for 10 min at 4 °C to remove insoluble material, and the cell-free supernatant was kept for analysis. The following analytes were investigated with a customized protein array (Quantibody^®^ Mutiplex ELISA Array, RayBiotech, GA, USA): C-reactive protein (CRP), immunoglobulin A (IgA), interleukin-6 (IL-6), interleukin-10 (IL-10), tumor necrosis factor-α (TNF-α), and nuclear factor kappa-light-chain-enhancer of activated B cells (NF-κB), according to the manufacturer’s instructions. In addition, alpha-amylase was examined with salivary α-amylase kinetic enzyme assay kit (Salmetrics^®^, PA, USA) per the manufacturer’s instructions.

### Statistical analysis

The clinical data of subjects were combined with PSR score and biomarker levels before analysis. All statistical analyses were performed with SPSS (IBM^®^ SPSS^®^ Statistics; Version 25). One-way ANOVA as well as Tukey’s post-hoc tests were performed to compare among three groups. The *p*-value less than 0.05 was considered as statistically significant. To identify significant individual identifiers of the different groups of H, DM, and DMN, the Bayes general linear model was performed. A binary response was used to look for variables that best separate two of the three groupings at a time. For the regression plot, the significance of the interaction term was calculated from an F-test. A PERMANOVA test was performed to determine the compositional differences between groups on analyte metrics using R package PERMANOVA. In addition, Spearman correlation analysis was performed to compare the analytes level in saliva.

## Supplementary Information

Supplementary Information.

## Data Availability

Our data are available per request to the corresponding author.
