# Supplementary material for: Changes in salivary biomarkers associated with periodontitis and diabetic neuropathy in individuals with type 1 diabetes
Source: Sci Rep. 2022 Jul 4;12:11284. doi: 10.1038/s41598-022-15430-0 (PMC9253002; doi:10.1038/s41598-022-15430-0)
Supplement: Supplementary file 1 — Supplementary Information. [file 41598_2022_15430_MOESM1_ESM.docx]

**Supplemental Data**

**Correlative changes in Salivary Analytes and Periodontitis related to Type I Diabetes-induced Neuropathy**

Running title: Saliva Analytes in Diabetes-induced Neuropathy

Larissa Steigmann^1,$^, Shogo Maekawa^1,2,3,$^, Frederic Kauffmann^1,4,$^, Jacob Reiss^5^, Ashley Cornett^6,7^, James Sugai^1,7^, Julian Venegas^8^, Xudong Fan^7,9^, Yuying Xie^8^, William V. Giannobile^1,2^, Rodica Pop-Busui^5^, Isabelle M.A. Lombaert^6,7^

^1^ University of Michigan, School of Dentistry, Department of Periodontics and Oral Medicine, Ann Arbor, MI 48106

^2^ Current address: Harvard School of Dental Medicine, Department of Oral Medicine, Infection, and Immunity, Boston, MA 02115

^3^ Tokyo Medical and Dental University, Department of Periodontology, Tokyo, Japan 113-8510

^4^ Department of Oral and Craniomaxillofacial Surgery, Center for Dental Medicine, University Medical Center Freiburg, Freiburg im Breisgau, Germany 79106

^5^ University of Michigan, School of Medicine, Department of Internal Medicine, Division of Metabolism, Metabolism Endocrinology and Diabetes, Ann Arbor, MI 48105

^6^ University of Michigan, School of Dentistry, Department of Biologic and Materials Sciences, Ann Arbor, MI 48109

^7^ University of Michigan, Biointerfaces Institute, Ann Arbor, MI 48109

^8^ Michigan State University, Department of Computational Mathematics, Science and Engineering, East Lansing, MI 48824

^9^ University of Michigan, College of Engineering, Department of Biomedical Engineering, Ann Arbor, MI 48109

**Supplementary Table 1.** **Enrollment scheme**.

| **Selection Inclusion Criteria** | |
| --- | --- |
| **Healthy** | Age matched healthy non-obese controls with normal glucose tolerance, normal blood pressure and cholesterol |
| **Diabetes** | Subjects with diagnosed diabetes as defined by the American Diabetes Association (Type I diabetes), and no complication of retinopathy, neuropathy, cardiovascular autonomic neuropathy, or mild-to-moderate peripheral neuropathy. |
| **Diabetes with Neuropathy** | Subjects with diagnosed diabetes as defined by the American Diabetes Association (Type I diabetes), and complications, including cardiovascular autonomic neuropathy and mild-to-moderate peripheral neuropathy. |
| **Selection Exclusion Criteria** | |
| **Diabetes** | Presence of any retinopathy, nephropathy, peripheral and autonomic neuropathy, and cardiovascular disease |
| **Diabetes with Neuropathy** | Any other causes of neuropathy besides diabetes (e.g. active hepatitis C, and stage renal disease, systemic lupus erythematous or a known hereditary neuropathy) as determined through medical history, family history, history of mediations, occupational history, history of exposure to toxins, physical and neurological examinations; |
|  | History of drug or alcohol abuse within the previous 2 years or current weekly alcohol consumption of > 10 units per week. |
|  | Participation in an experimental medication trial within 3 months |

Table represents the inclusion and exclusion selection for the 3 different groups (Healthy/Diabetes/Diabetes with Neuropathy).

**Supplementary Table 2. Demographic and periodontal parameters of the three experimental groups.**

| **Demographics** | | | **Healthy** | **Diabetes** | **Diabetes** | **Diabetes with Neuropathy** | **Diabetes with Neuropathy** |
| --- | --- | --- | --- | --- | --- | --- | --- |
|  |  |  |  | **+Healthy Perio** | **+Gingivitis** | **+healthy Perio** | **+Gingivitis** |
|  | N (%) |  | 10 | 6 | 3 | 5 | 6 |
|  | Age (yrs) |  | 40 ± 12 | 50 ± 14 | 35 ± 17 | 54 ± 13 | 56 ± 10 |
| **Clinical Measurements** | | |  |  |  |  |  |
|  |  | Weight (kg) | 71.6 ± 14.8 | 78.1 ± 23.8 | 97.2 ± 15.3 | 91.3 ± 8.2 | 91.1 ± 17.0 |
|  |  | Height (m) | 1.72 ± 0.13 | 1.69 ± 0.10 | 1.79 ± 0.08 | 1.74 ± 0.09 | 1.77 ± 0.04 |
|  |  | BMI | 24.0 ± 2.8 | 26.9 ± 5.1 | 30.3 ± 4.9 | 30.4 ± 4.1 | 29.2 ± 5.6 |
|  |  | HbA1c | 5.4 ± 0.1 | 7.5 ± 0.9 | 10.0 ± 3.5^a^ | 8.5 ± 1.6 | 8.3 ± 1.8 |
|  |  | Fasting Glucose | 85.0 ± 10.1 | 150.2 ± 53.8 | 212.7 ± 142.5 | 176.3 ± 64.9 | 132.0 ± 81.4 |
|  |  | Duration of Disease | 0 | 18.5 ± 6.2^a^ | 11.7 ± 7.5 | 33.8 ± 18.1^aa,d^ | 31.7 ± 12.7^aa^ |
|  |  | Amount of Smoking (pack / year) | 0.11 ± 0.33 | 0.60 ± 1.34 | 0.0 ± 0.0 | 6.25 ± 12.50 | 0.0 ± 0.0 |
|  |  | mean PSR | 0.30 ± 0.42 | 0.33 ± 0.52 | 1.89 ± 0.19^aa,bb,cc^ | 0.0 ± 0.0 | 1.83 ± 0.33^aa,bb,cc^ |
|  |  | α-Amylase level (U/ml) | 104.0 ± 91.8 | 166.9 ± 130.7 | 162.5 ± 117.6 | 163.5 ± 137.5 | 106.4 ± 104.0 |
|  |  | CRP | 763.1 ± 799.2 | 507.4 ± 296.9 | 855.7 ± 415.7 | 577.9 ± 250.6 | 866.4 ± 376.2 |
|  |  | IgA | 17182.2 ± 8899.3 | 9660.8 ± 5702.9 | 6882.8 ± 1847.1 | 5905.0 ± 3124.8^a^ | 16894.6 ± 7084.3 ^p=0.059^ |
|  |  | IL-10 | 6.5 ± 7.7 | 2.8 ± 1.7 | 2.2 ± 1.1 | 4.1 ± 2.6 | 13.8 ± 19.6 |
|  |  | IL-6 | 409.6 ± 493.5 | 375.6 ± 287.7 | 894.6 ± 898.6 | 601.3 ± 271.6 | 967.3 ± 1248.2 |
|  |  | NFkB | 2423.4 ± 1602.5 | 1775.3 ± 1331.8 | 2596.0 ± 1241.1 | 3304.5 ± 667.9 | 6676.9 ± 8320.0 |
|  |  | TNF-α | 1893.4 ± 3027.6 | 1308.3 ± 893.8 | 752.1 ± 174.3 | 1483.7 ± 523.0 | 4987.5 ± 7527.6 |

Sociodemographic parameters and saliva biomarker levels are shown. Data are presented as mean ± standard deviation. Tukey test (for continuous data) was used to assess differences between groups. a) significantly different from Healthy, a: p < 0.05, aa: p < 0.01. b) significantly different from Diabetes+Healthy Perio, bb: p < 0.01. c) significantly different from Diabetes with Neuropathy+Healthy Perio, cc: p< 0.01. d) significantly different from Diabetes+Gingivitis, d: p< 0.05.

**
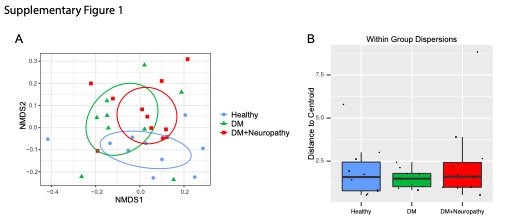
**

**Supplementary Figure 1. Subject population analysis.**

1. Non-metric multi-dimensional scaling (NMDS) of all the systemic features and the saliva analytes.
2. a box plot of the distances of the features from the respective group centroids.

**Supplementary Figure 2. Trend in correlation of the systemic features and analytes among the group.**

The correlation values were represented in line graphs. There were 5 trends, the line looks Flat (black), V shape (blue), Inverted V shape (green), Increasing (Red), Decreasing (Pink).

**Supplementary Figure 3. The interaction regression plot using IgA and α-Amylase.**

The p-value was corresponding to an F-test from ANOVA model comparison.
